# Regulation of intestinal microflora and metabolites of *Penthorum chinense* Pursh on alcoholic liver disease

**DOI:** 10.3389/fphar.2023.1331956

**Published:** 2024-01-24

**Authors:** Hui Zhang, Xiao Cui, Wei Liu, Zheng Xiang, Ji-Feng Ye

**Affiliations:** ^1^ Department of Pharmacy, The Second Affiliated Hospital and Yuying Children’s Hospital of Wenzhou Medical University, Wenzhou, China; ^2^ School of Pharmaceutical Science, Liaoning University, Shenyang, China

**Keywords:** Penthorum chinense pursh, alcoholic liver disease, gut microflora, metabolomics, UPLC-MS

## Abstract

**Introduction:** Alcoholic liver disease (ALD) was the second leading cause of liver injury. *Penthorum chinense* Pursh (GHC) is an important *Miao* ethnic drug of traditional Chinese medicine for the treatment of liver disease, but the pathogenesis is not clear.

**Aim of the study:** To analysis the intestinal microflora and metabolic pathway of GHC on ALD mice.

**Methods:** An HPLC-QTOF-MS method was used to identified the components from GHC extract, firstly. 60 mice were divided into six groups including blank group, model group, positive group and GHC groups (0.29, 0.87 and 2.61 g/kg). ALD mice was treated with GHC for 12 days. ALT, AST, TC and TG in serum were determined, liver index and pathological analysis were achieved. 16S rRNA gene sequencing was used to detect the intestinal microbial diversity. Finally, UPLC-QTOF-MS was used to analysis the metabolic pathways.

**Results:** 38 ingredients were identified in GHC extract. Compared with the model group, liver index of the positive group and GHC (2.61 g/kg) group was significantly reduced. Compared with the model group, contents of ALT, AST, TC and TG of GHC groups reduced in a dose-dependent manner. Intestinal microbial diversity analysis indicated that Chao1, Observed species, Pielou_e, and Shannon indexes in GHC group (2.61 g/kg) were lower than those in model group. Principal coordinate analysis indicated that the intestinal microbial composition between blank group and model group, the model group and GHC (2.61 g/kg) group changed significantly. Compared with the model group, proportion of *Firmicutes* decreased, and the proportion of *Bacteroidetes* increased significantly in GHC group, which were 50.84% and 40.15%. The more prominent bacteria in the GHC group were *odoribacteraceae*, *turicibacter*, *deferribacteraceae*, and the intestinal beneficial symbiotic bacteria mucispirillum. Metabolic analysis indicated that, compared with blank group, 90 metabolites in model group changed significantly, and 68 metabolites were significantly callback in GHC group.

**Discussion:** GHC has a therapeutic effect on ALD by regulating intestinal flora imbalance and metabolic pathways including Glycine, serine and threonine metabolism, Glutathione metabolism, Arginine and proline metabolism, Alanine, aspartate and glutamate metabolism, Butanoate metabolism and primary bile acid biosynthesis.

## 1 Introduction

Alcoholic liver disease (ALD) is a common liver disease caused by long-term excessive drinking, and it is also the main cause of cirrhosis and hepatogenic death. ALD often occurs in alcoholics, which can induce alcoholic hypoglycemia, intrahepatic cholestasis, high-fat hemolysis syndrome, and even liver failure (Lee et al., 2020). ALD includes alcoholic fatty liver (AFL), alcoholic hepatitis (AH), alcoholic hepatic fibrosis (AHF) and alcoholic cirrhosis (AC) (Wang, 2021). ALD has become the second leading cause of liver injury with the consumption of alcohol increased (Wilfred de Alwis and Day, 2007). The pathogenesis of ALD is not fully understood. It is currently believed that oxidative stress, steatosis, intestinal microflora imbalance and inflammatory mediator damage are the main causes of ALD (Gao and Bataller, 2011; Osna et al., 2017; Woodhouse et al., 2018; Zhao et al., 2018).

Gut microbiota is a microbial population that parasitizes in the intestinal tract. It is one of the most dense and active microbial ecosystems. Gut microbiota can provide necessary nutrients such as vitamins, enzymes and fatty acids required by the body, and affect lipid metabolism, host homeostasis, bile acid metabolism and intestinal barrier maintenance through metabolic activities and host interactions (Rowland et al., 2018; Di Meo et al., 2019; Lavelle and Sokol, 2020; Agus et al., 2021). The gut microbiota is one of the key participants in ALD. Microbial dysbiosis is a characteristic of liver diseases, including ALD. Excessive alcohol consumption could leads to the destruction of the intestinal epithelial barrier, increased permeability of the intestinal wall, and the entry of a large amount of endotoxin into the circulatory system (Scott, 2017). Bacterial endotoxin LPS is a prototype microbial derived inflammatory signal that can promote inflammation of ALD by activating Toll like receptor 4 (TLR4) (Szabo, 2014). Therefore, treating ALD by focusing on gut microbiota is an important clinical application method.

Bile acids (BAs) and gut microbiota play a crucial role in the pathogenesis of ALD through the gut microbiota-bile acids-liver axis (Freeman et al., 2005). Bile acids are the most important components of the intestinal chemical barrier, which can promote the absorption of food, but also help the body to maintain intestinal mucosal function and intestinal environment stability (Wang et al., 2015). In ALD patients, the transport and homeostasis of BAs are destroyed, resulting in cholestasis, which is a key pathogenic factor of ALD. Therefore, the regulation of bile acid metabolism or related signaling pathways is increasingly considered as a potential therapeutic strategy for ALD (Liu et al., 2022a).

The interaction between bile acids and intestinal flora is bidirectional. Bile acids can reshape the structure of intestinal flora. And intestinal flora also has a regulatory effect on bile acids (Di Ciaula et al., 2017; Shao et al., 2021). Bile acids have antibacterial properties, which can regulate the structure of intestinal flora by destroying the intestinal mucosal cell barrier or interfering with the intestinal immune response. In addition, it can also induce the production of antibacterial peptides through FXR and induce the host immune response through FXR to indirectly affect the signal characteristics or composition of the flora (De Waziers et al., 1995; Song et al., 2019). Alcohol increases the size of bile acid pools and reduces fecal bile acid excretion, resulting in specific changes in the gut microbiota (Chiang and Ferrell, 2019; Ciocan et al., 2019; Yu et al., 2020). These findings suggested that alcohol-induced bile acid-intestinal flora axis imbalance may be a potential pathophysiological mechanism of ALD. Clinically, glucocorticoids and silymarin are usually used to treat ALD. However, some patients have hormone tolerance and has obvious side effects (Deng et al., 2015). Therefore, it is imperative to find safer and more effective drugs for the treatment of ALD.

*Penthorum chinense* Pursh (Ganhuang Cao, GHC) is an important *Miao* ethnic drug of traditional Chinese medicine for the treatment of liver disease in the *Miao* ethnic area of Sichuan Province, China (Zhang et al., 2013). In the theory of Chines Medicine, it has the effects of removing jaundice and dampness, clearing heat and detoxifying. Gansu Granule, a kind of granule made from GHC was used in the treatment of chronic active hepatitis, hepatitis B and acute viral hepatitis clinically. In recent years, the efficacy of GHC in the treatment of ALD has been reported (Lei et al., 2018; Jiu et al., 2023), but the mechanism is less studied. In this study, the ALD mice were established to evaluate the therapeutic effect of GHC. Subsequently, the mechanism of GHC in the treatment of ALD was analyzed by the regulation of gut microbiota and metabolomics.

## 2 Materials and methods

### 2.1 Drugs and reagents

*Penthorum chinense* Pursh was purchased from Chengdu Deng’s Chinese herbal medicine Co. (Chengdu, China), Kits for determining the total cholesterol (TC), triglyceride (TG), aspartate amino transferase (AST), and alanine aminotransferase (ALT) were purchased from Nanjing Jiancheng Bioengineering Institute (Nanjing, China). Normal saline was obtained from Harbin Sanlian Pharmaceutical Co., Ltd (Harbin, China). Tissue fixative was purchased from Wuhan Servicebio Technology Co., Ltd (Wuhan, China). Silybin was purchased from Shanghai Aladdin Biochemical Technology Co., Ltd (Shanghai, China). Alcohol was purchased from Beijing Red Star Co., Ltd (Beijing, China). Carbamazepine and mycophenolic acid were purchased from Anpel Laboratory Technologies (Shanghai) Inc. (Shanghai, China).

### 2.2 Animals

60 male mice weighing 18–22 g were purchased from Liaoning Changsheng Biotechnology Co., Ltd. During the experiment, the mice were kept in the animal room of School of Pharmaceutical Science in Liaoning University, with a relative humidity of 45% ± 10% and a temperature controlled at 22 ± 2°C, with a light/dark cycle for 12 h. The experimental process was approved by the Institutional Animal Ethics Committee of Liaoning University.

### 2.3 Chemical composition determination of the extract from the GHC

#### 2.3.1 Preparation of the test sample

After vacuum drying, 2 g of GHC were accurately weighed and placed in a conical flask, added with 20 mL of water, and ultrasonically extracted at room temperature for 30 min. The extract was filtered through filter paper and concentrated to obtain extract. The extract was dissolved in 70% methanol solution and diluted to 100 mL. The 2.0 mL solution was filtered through 0.22 μm microporous membrane for later use.

#### 2.3.2 UPLC-MS analysis

An HPLC-QTOF/MS method was established to identify the chemical components in the extract of GHC. An Acquity UPLC BEH C18 column was used to carry out separation alone with a mobile phase consisting of acetonitrile (A) and water containing 0.1% formic acid (B). The gradient elution was as follows: 0–90 min, 5%–45% (A); 90–95 min, 45%–5% (A). The injection volume was 10 μL, the temperature of the column was 35°C, and the flow rate was 0.6 mL/min.

### 2.4 Preparation of GHC extract

Dried leaves of *Penthorum chinense* Pursh (1.5 kg) was soaked in water (15 L) for 0.5 h, refluxed for 1 h and filtered, the residue was then added with water (12 L), refluxed for 0.5 h and filtered, after the supernatant was combined and then concentrated under vacuum to obtained the extract. The extract was diluted with water to a concentration of 0.106 g/mL and stored at 4°C before use.

### 2.5 Induction of ALD and treatment

After adaptive feeding for 7 days, 60 male mice were randomly divided into 6 groups (*n* = 10) including blank group, model group, positive group (silybin, 50 mg/kg) and GHC groups with three dosage (0.29, 0.87, 2.61 g/kg). In the GHC groups, the mice were given the GHC by gavage for 7 consecutive days, and the blank group and the model group were given the same volume of normal saline. From the 7^th^ day, after 4 h of GHC was given, the mice in the model group, positive group and GHC groups were given 56°liquor (13 mL/kg) by gavage for 5 consecutive days to evaluate the protective effect of GHC on ALD. The blank group was given the same volume of normal saline. On the 12th day, all the mice were euthanized with pentobarbital sodium (50 mg/kg, IP), blood samples were taken from mice and centrifuged at 3,500 rpm for 10 min to obtain serum. Serum samples were used for metabolomics analysis and determination of TC, TG, ALT and AST. Liver tissue was obtained and washed with normal saline. The normal saline was dried with a clean filter paper and the weight of liver was measured to calculate the liver index. Liver index = (liver weight/body weight × 100%). Part of the liver tissue was fixed to make pathological analysis. The intestinal feces of mice were collected under sterile conditions, and stored at −80°C for intestinal flora analysis.

### 2.6 16S rRNA gene sequencing

The OMEGA Soil DNA Kit (M5635-02) (Omega Bio-Tek, Norcross, GA, United States) was used to isolate total bacterial DNA from fecal samples. The molecular size was determined by 0.8% agarose gel electrophoresis, and the DNA was quantified by nanodroplet NC2000 spectrophotometer (Thermo Fisher Scientific, Waltham, MA, United States). Bacterial 16S rDNA V3-V4 region specific primers (upstream primer: 5′-ACT CCT ACG GGA GGC AGC A-3′, downstream primer: 5′-GGA CTA CHV GGG TWT CTA AT-3′) were selected for PCR amplification. The PCR products were quantified on a Microplate reader (BioTek, FLx800) using Quant-iT PicoGreen dsDNA Assay Kit, and then mixed according to the amount of data required for each sample. The sequencing service was provided by Shanghai Personalbio Technology Co., Ltd. The α diversity, including indices such as Chao1 and Shannon, was calculated using the operational taxonomic units (OTUs) in QIME. The β diversity was visualized by PCoA.

### 2.7 Metabolic analysis

GHC exhibited therapeutic effects on ALD with a dosage-dependent manner. So, serum of blank group, model group and GHC (2.61 g/kg) group were used for metabolic analysis. Briefly, 150 μL of serum was accurately absorbed, 150 μL of methanol including internal standards (carbamazepine and mycophenolic acid, 50 ng/mL, respectively) and 600 μL of methanol was added, vortexed and mixed for 30 s, stored at 4°C for 30 min. After centrifuging at 12,000 rpm for 15 min (4°C), 700 μL of the supernatant was taken into a tube, dried by nitrogen, and stored in a refrigerator at −80°C. Before analysis, the sample was redissolved with 70 μL of 5% acetonitrile aqueous solution, centrifuged at 12,000 rpm for 10 min (4°C), and 50 μL of supernatant was accurately added into the sample bottle.

Mass spectrometry was performed on an AB X500R QTOF LC-MS/MS system. The samples were separated on an Exion LC equipped with an ACQUITY UPLC ^®^ BEH C18 (1.7 μm, 2.1 × 100 mm) column. Gradient elution was performed with water (A) and methanol (B) as the elution system.: 0–5 min 70% (B), 5–13 min 70%–98% (B), 13–15 min 98% (B), 15–15.1 min 98%–50% (B), 15.1–18 min 50% (B). The injection volume was 10 μL, the elution flow rate was 0.3 mL/min, and the column temperature was 30°C.

### 2.8 Statistical analysis

GraphPad Prism 5.0 software was used to analyze the data. Statistical analysis was performed using one-way analysis of variance (ANOVA) and Tukey’s multiple comparison test. All experimental data were expressed as mean ± SD, and *p* < 0.05 was considered statistically significant.

## 3 Results

### 3.1 UPLC-MS analysis of component identification in GHC

**FIGURE 1:**
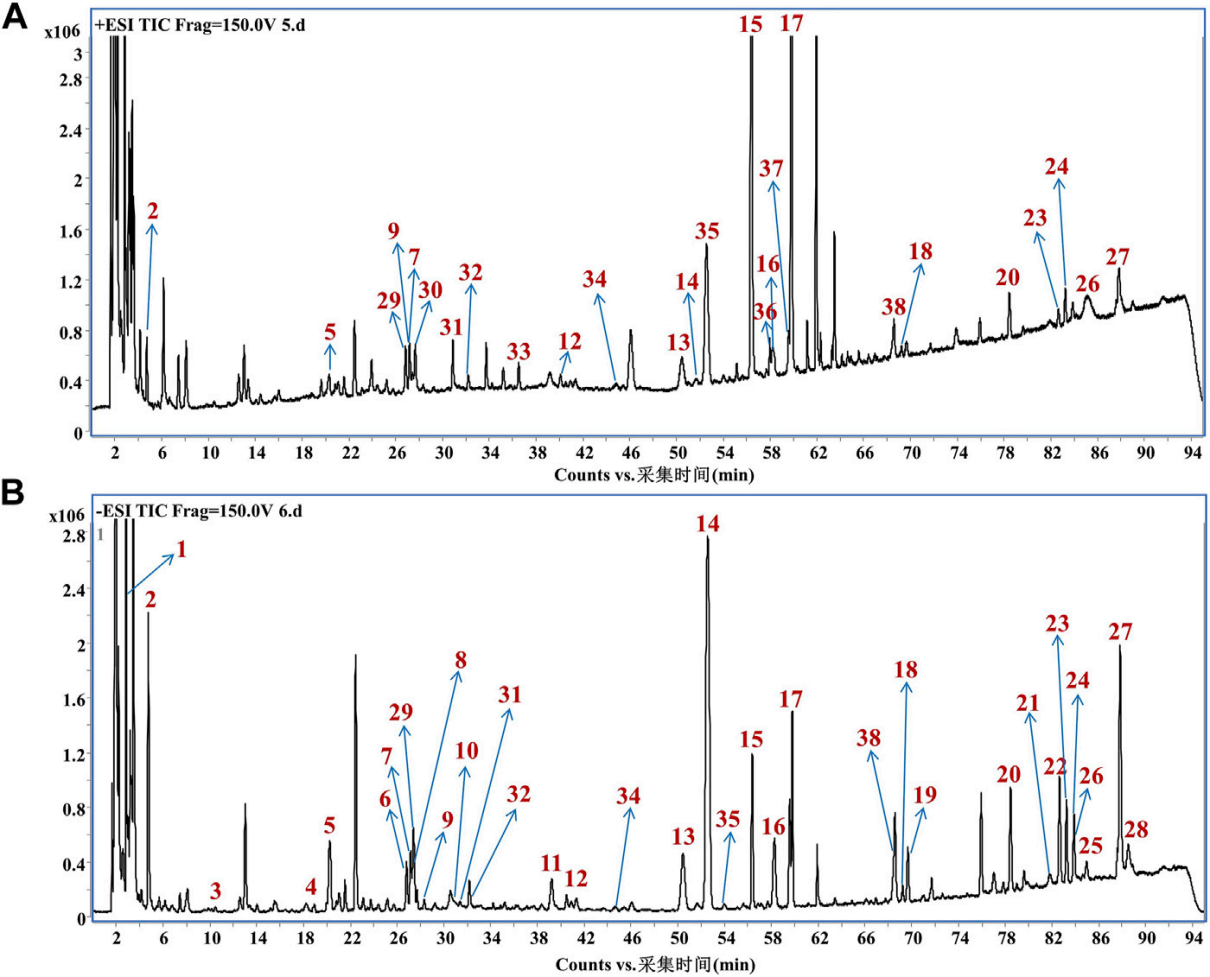
Mass spectrum of chemical constituents in GHC extracts. **(A)** Total ion chromatogram in positive ion mode; **(B)** Total ion chromatogram in negative ion mode.

The chemical constituents of GHC were analyzed by HPLC-QTOF/MS. A total of 38 chemical constituents were identified, including 7 organic acids, 26 flavonoids and 3 lignans (Figure 1; Supplementary Table S1).

### 3.2 Effects of GHC on body weight of mice

**FIGURE 2:**
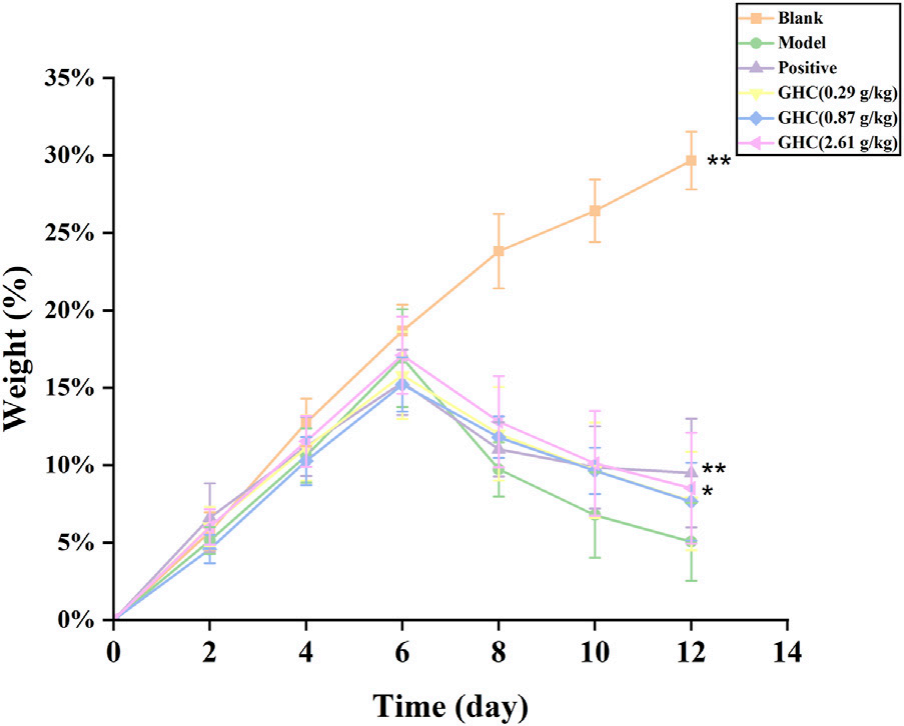
Effect of *Penthorum chinense* Pursh on body weight of mice. * Compared with the model group, * *p* < 0.05, ** *p* < 0.01.

During the experiment, the mice were weighed every day, and the percentage of increased weight of the mice was calculated. The results showed that from the 7^th^ day, the increased weight of the mice in the model group was significantly lower than that in the blank group. The increased weight percentage of mice in the positive drug group and the GHC (2.61 g/kg) group on the 12th day was significantly higher than that in the model group (Figure 2).

### 3.3 Effect of GHC on liver index in mice

**FIGURE 3:**
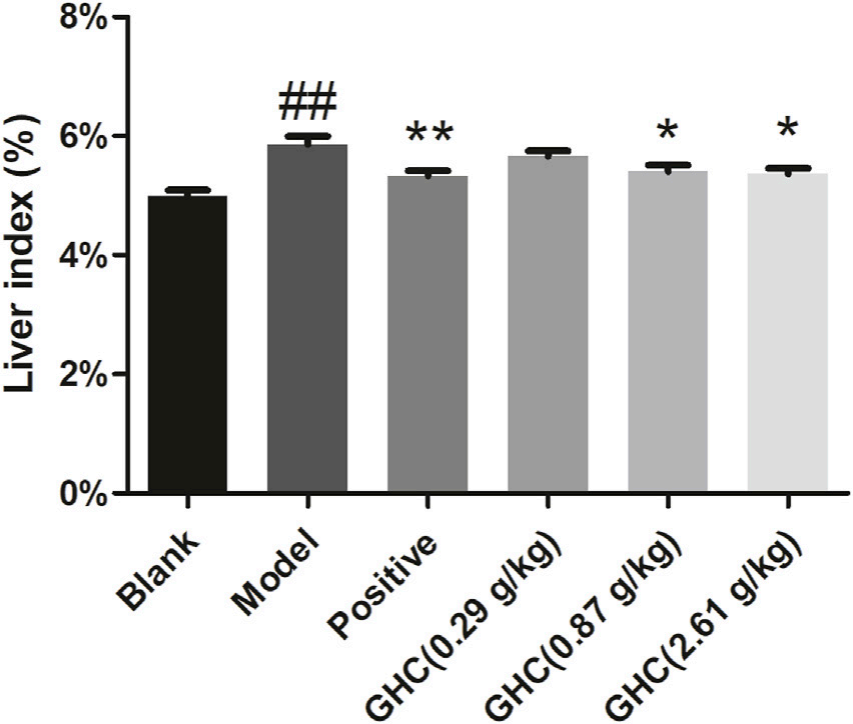
Effect of *Penthorum chinense* Pursh on liver index in mice. * Compared with the model group, * *p* < 0.05, ** *p* < 0.01; # Compared with the blank group, # *p* < 0.05, ## *p* < 0.01.

Liver index of the model group was significantly higher than that of the blank group (*p* < 0.01), indicating that the ALD model was established. Compared with the model group, the liver index of the positive group and the GHC (2.61 g/kg) group was significantly reduced (*p* < 0.05), indicating that GHC can effectively treat alcohol-induced liver edema and reduce alcohol damage to the liver (Figure 3).

### 3.4 Effects of GHC on serum TC, TG, ALT and AST levels in mice

**FIGURE 4:**
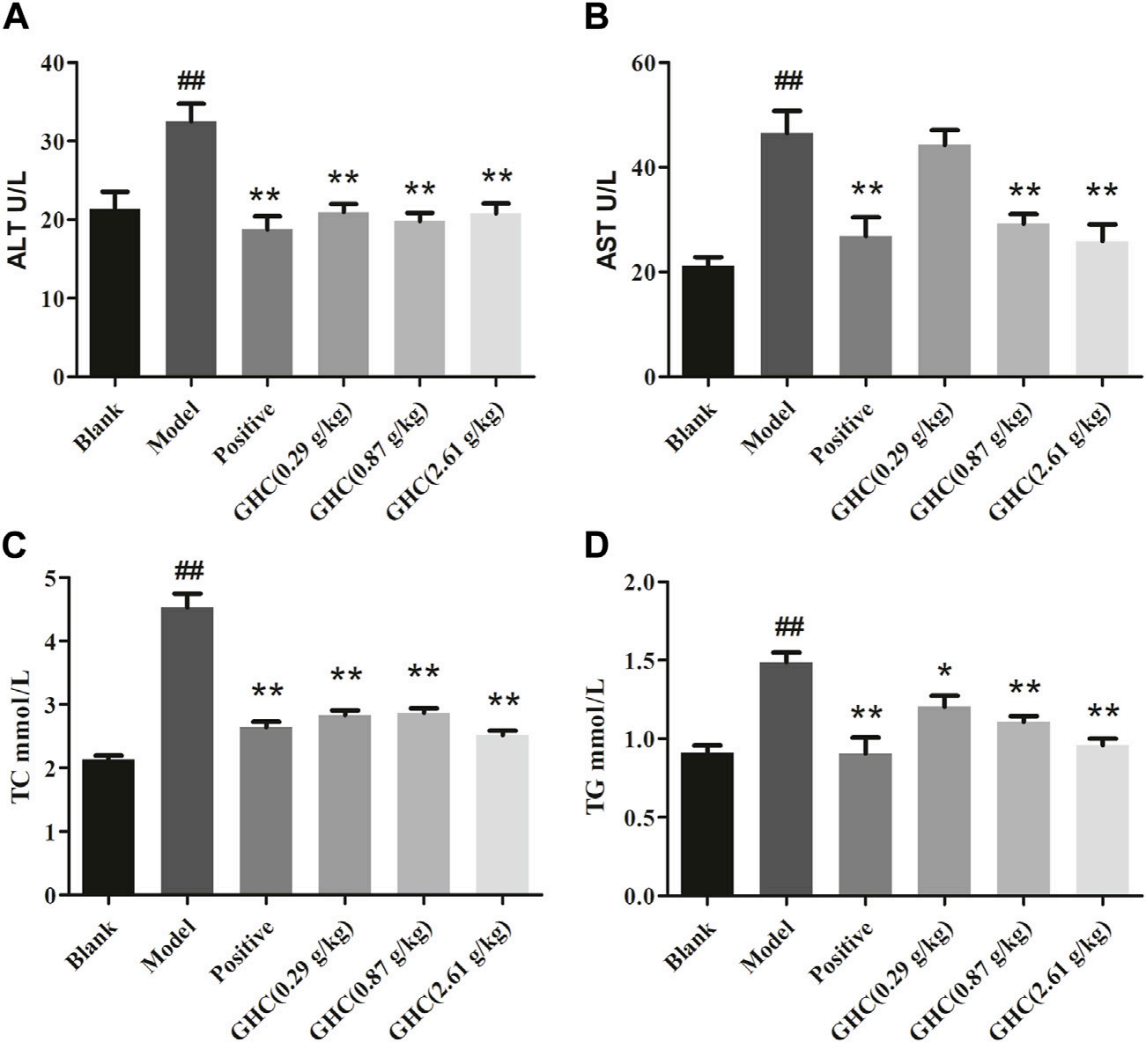
Effects of *Penthorum chinense* Pursh on ALT, AST, TC and TG in serum of mice with alcoholic liver injury. **(A)** ALT, **(B)** AST, **(C)** TC, **(D)** TG. * Compared with the model group, * *p* < 0.05, ** *p* < 0.01; # Compared with the blank group, # *p* < 0.05, ## *p* < 0.01.

ALT and AST in serum could directly reflect the degree of liver injury in mice, TG and TC can show the blood lipid content of mice (Figure 4). Compared with the blank group, the contents of ALT, AST, TC and TG in the serum of the model group were increased (*p* < 0.01, *p* < 0.05), indicating that the liver function damage and fat accumulation in model group. The positive group and the GHC groups could reduce the contents of ALT, AST, TC, and TG in serum in a dose-dependent manner (*p* < 0.05).

### 3.5 Pathological analysis

**FIGURE 5:**
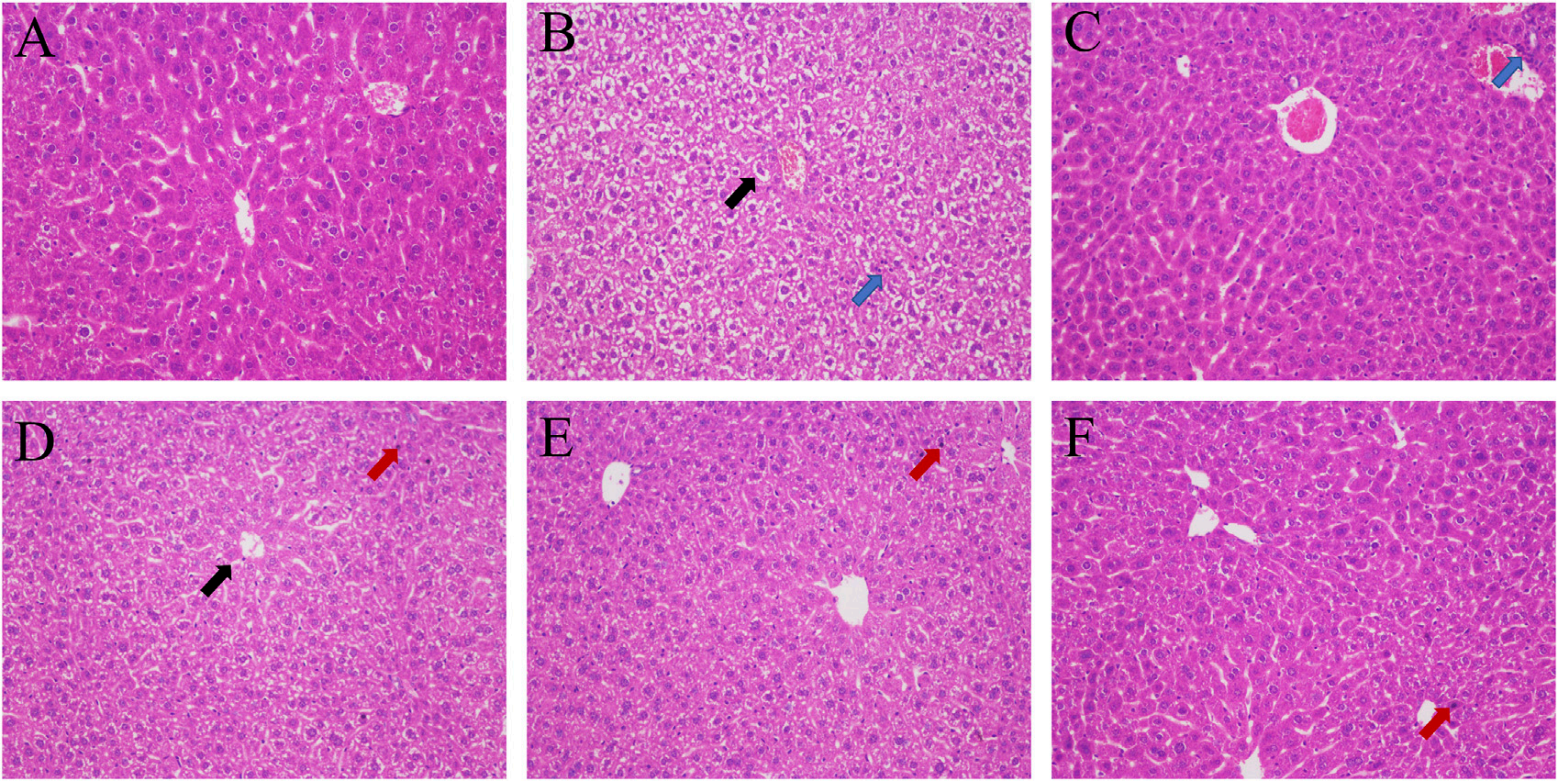
HE staining of liver tissue (200 ×). **(A)** blank group; **(B)** model group; **(C)** positive group; **(D)** GHC (0.29 g/kg) group; **(E)** GHC (0.87 g/kg) group; **(F)** GHC (2.61 g/kg) group (black → means vacuolization, red → means nuclear pyknosis, blue → inflammatory infiltration).

Hepatic lobule structure of the blank group was very clear, the boundary plate was neat, the cell cords were arranged in an orderly manner, the cell structure was clear, there was no foamy variation, and there was no congestion in the hepatic sinus. In the model group, balloon-like degeneration of hepatocytes, light staining of cytoplasm and infiltration of some inflammatory cells occurred. In the GHC groups and the positive group, the turbidity and swelling of hepatocytes and the disorder of hepatocyte cords were improved significantly. The boundary of hepatic lobules also became obvious, and no obvious cell necrosis was found (Figure 5).

### 3.6 Effect of GHC on intestinal flora biodiversity in mice

16S rRNA sequencing analysis was used to determine the changes of intestinal flora in blank group, model group and GHC (2.61 g/kg) group.

#### 3.6.1 Alpha diversity analysis

**FIGURE 6:**
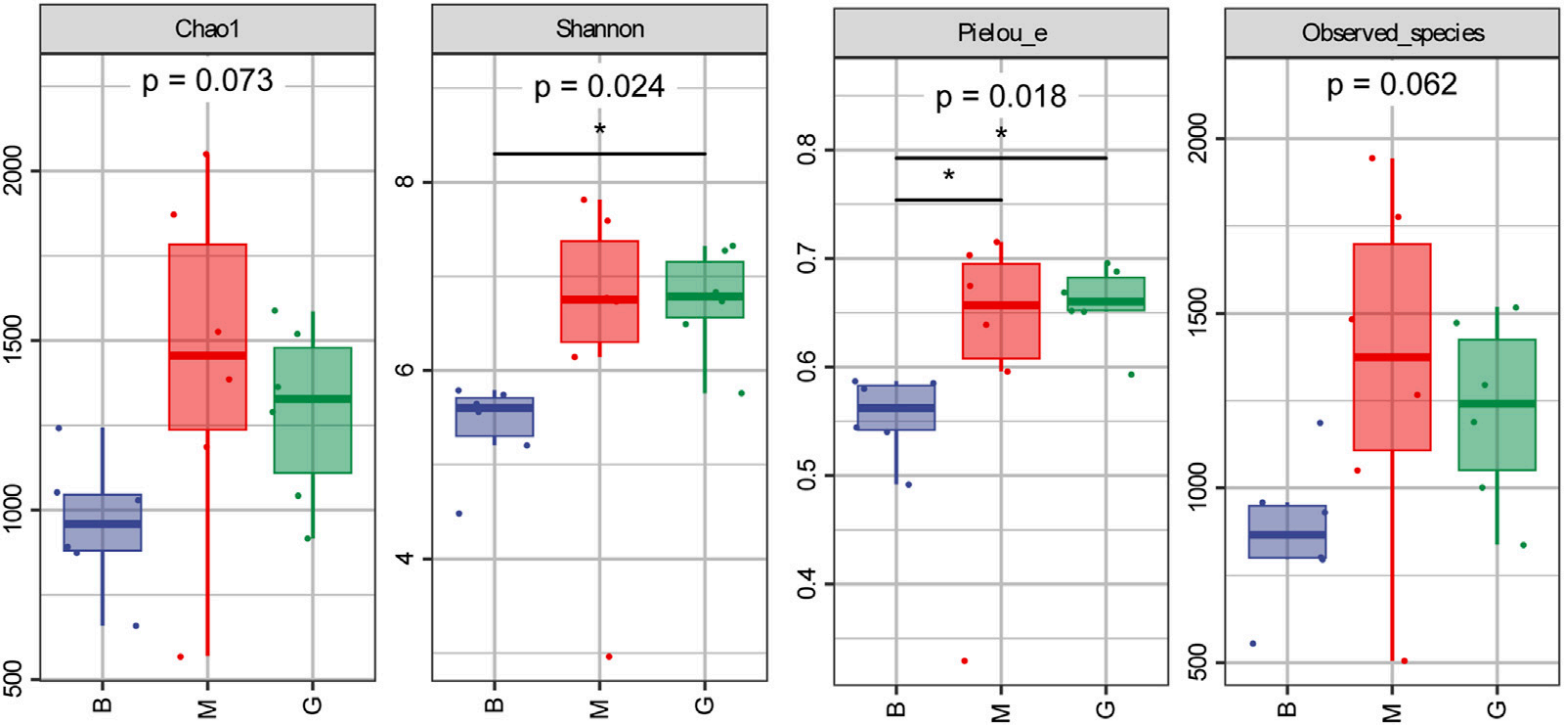
Alpha diversity index.

Compared with the blank group, the intestinal Chao1, Observed species, Pielou _ e and Shannon indexes of the model group were increased. The Chao1, Observed species, Pielou _ e, and Shannon indexes of the intestinal tract in the GHC (2.61 g/kg) group were lower than those in the model group (Figure 6).

#### 3.6.2 Beta diversity analysis

**FIGURE 7:**
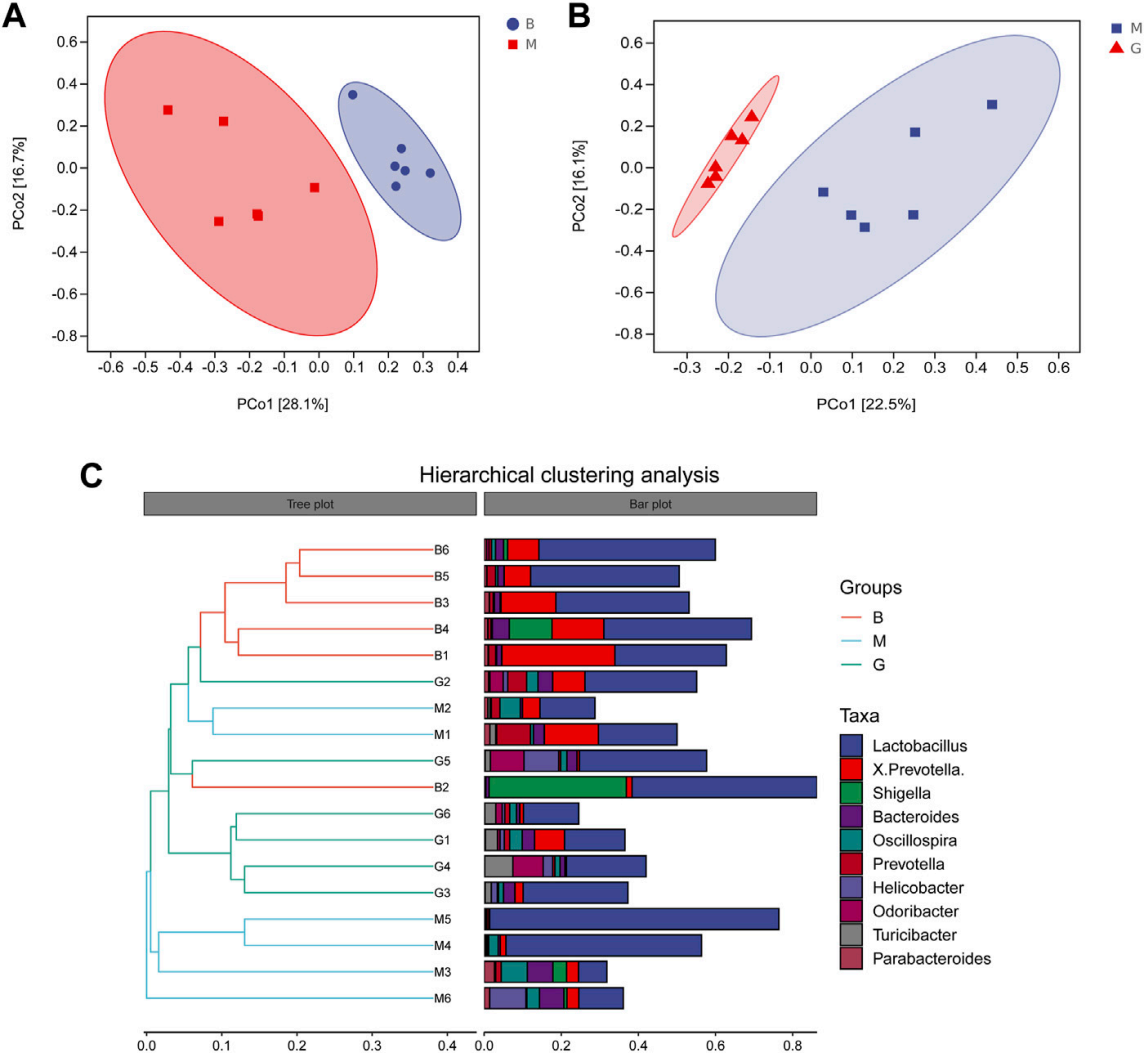
Beta diversity analysis. **(A)** PCoA analysis Blank group vs. Model group; **(B)** PCoA analysis GHC group vs. Model group; **(C)** Hierarchical clustering analysis.

Principal coordinate analysis (PCoA) was used to reduce the dimension of multidimensional microbial data, and the main trend of data change was demonstrated by the distribution of samples on the continuous sorting axis. Clustering analysis was used to identify the subset of discontinuous objects in the environment and classify the data. The results showed that between the blank group and the model group (Figure 6A), between the model group and the GHC (2.61 g/kg) group (Figure 6B), the samples were significantly separated without aggregation, indicating that the intestinal microbial composition between the groups changed significantly (Figure 7).

#### 3.6.3 Species composition analysis of intestinal flora

**FIGURE 8:**
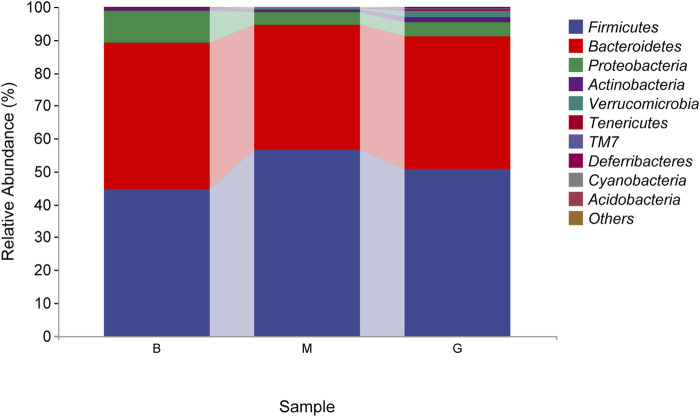
Taxonomic composition analysis (phylum level).

The species composition of intestinal flora in each group were dominated by Firmicutes and Bacteroidetes at the phylum level. The proportion of Bacteroidetes in the blank group was 44.80%, which was the absolute dominant phylum, and Firmicutes accounted for 44.50%. Compared with the blank group, the proportion of Firmicutes in the model group increased, and the proportion of Bacteroidetes decreased, which were 56.42% and 38.14%, respectively. After the administration of *Penthorum chinense* Pursh, compared with the model group, the proportion of Firmicutes decreased, and the proportion of Bacteroidetes increased significantly, which were 50.84% and 40.15%, respectively (Figure 8).

#### 3.6.4 Differences analysis of bacterial species

**FIGURE 9:**
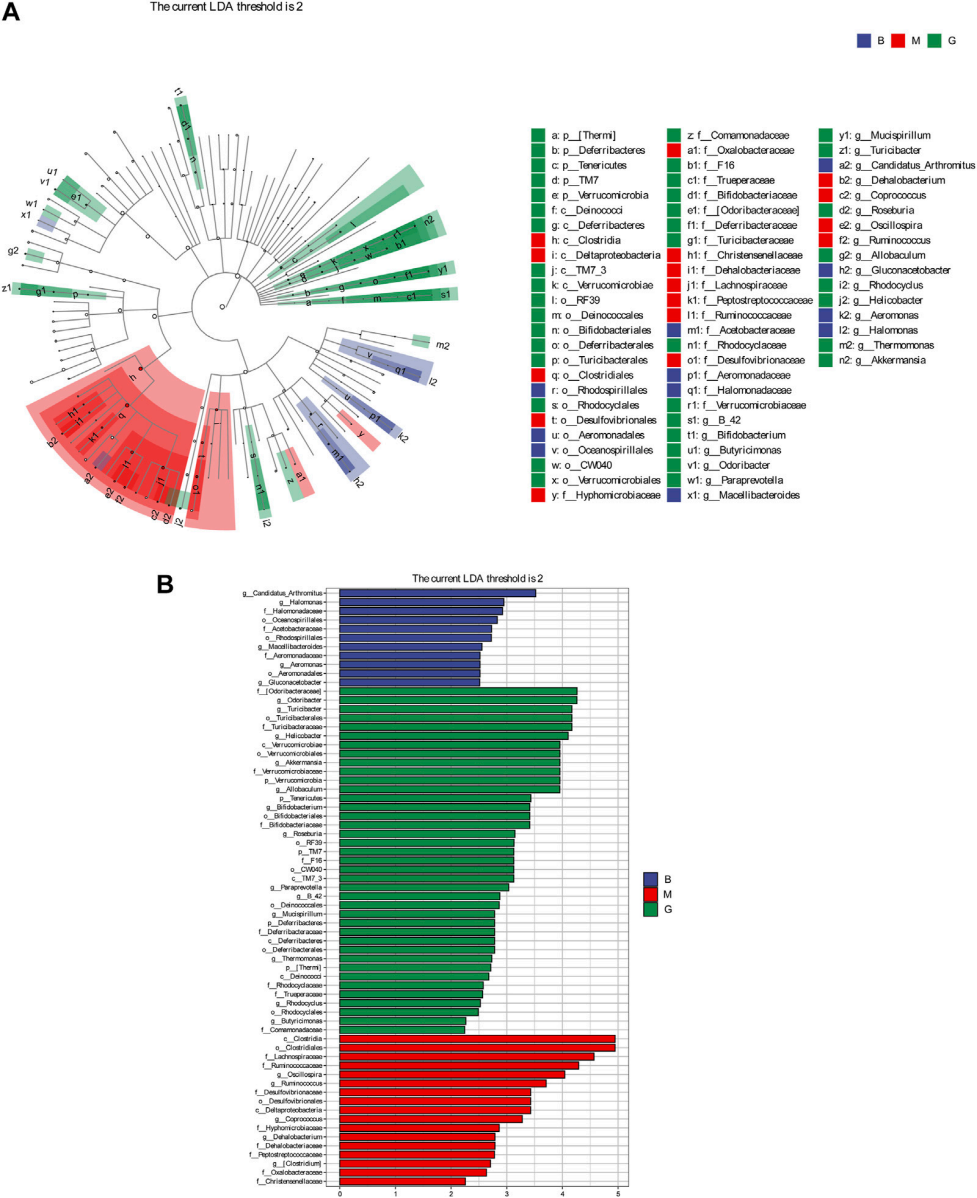
Comparison of significantly different intestinal flora between groups. **(A)** LEfSe evolutionary branch diagram, **(B)** LEfSe statistical difference analysis histogram.

LEFSe analysis is to detect the different strains between groups. Linear discriminant analysis (LDA) was used to estimate the species with significant differences between groups, and to determine the effect on the difference of species richness in each group. Combined with the results of the evolutionary branch diagram, a total of 67 different levels of taxa were identified, which had different degrees of richness in the blank group, model group and GHC group (Figure 9). Candidatus _ Arthromitus, Halomonas and Macellibacteroides were the main differential bacteria in the blank group, while oscillospira, ruminococcus and coprococcus were the main differential bacteria in the model group. The more prominent bacteria in the GHC group were odoribacteraceae, turicibacter, deferribacteraceae, and the intestinal beneficial symbiotic bacteria Mucispirillum.

### 3.7 Metabolic analysis

**FIGURE 10:**
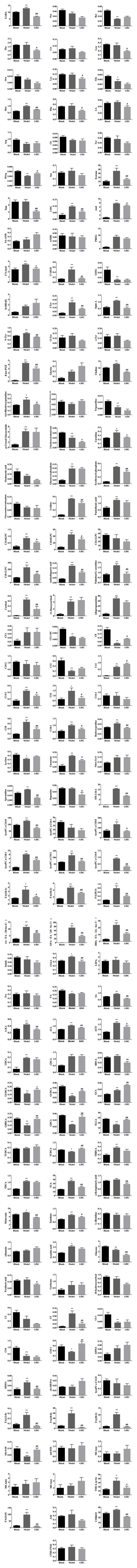
Effect of Penthorum chinense Pursh on serum metabolites in mice with acute alcoholic liver injury. * Compared with the blank group, * *p* < 0.05, ** *p* < 0.01; # Compared with the model group, # *p* < 0.05, ## *p* < 0.01.

The identification of metabolites was performed by comparing with the standard compounds. The changes of serum metabolites in each group were analyzed (Supplementary Figure S1). Compared with the blank group, 90 metabolites in the model group changed significantly, and 68 metabolites were significantly callback after the administration of *Penthorum chinense* Pursh (Figure 10).

**FIGURE 11:**
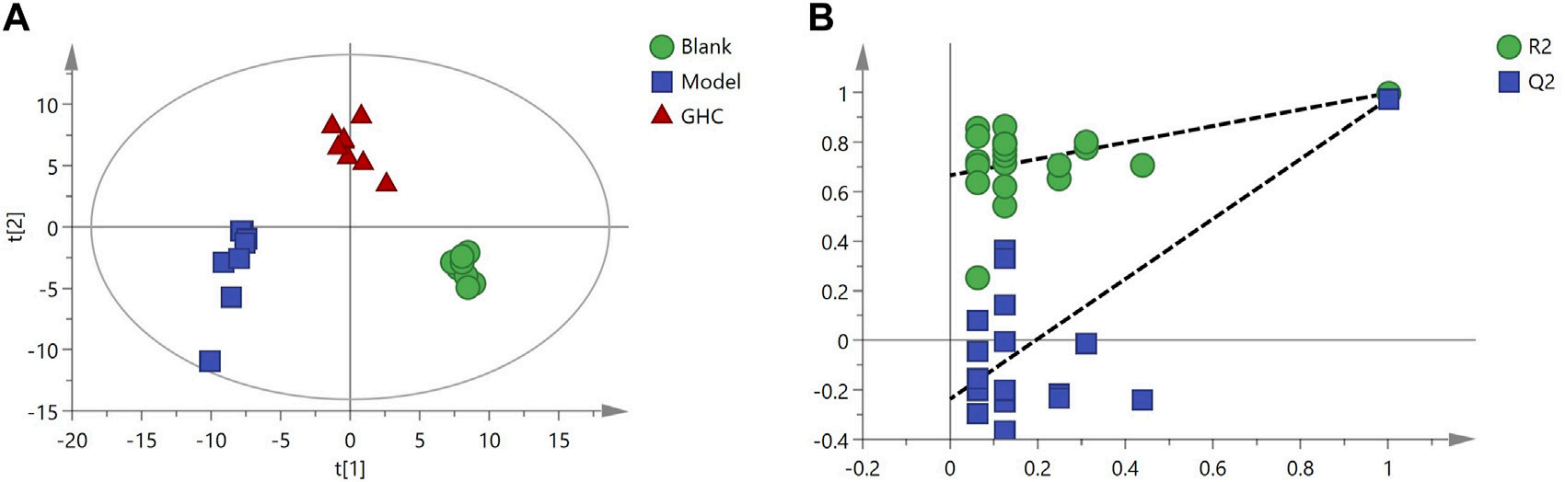
PLS-DA score plot and P-test plot of serum metabolites. **(A)** PLS-DA, **(B)** P-test.

Multivariate statistical analysis was performed on serum metabolite data of blank group, model group and GHC group. PLS-DA analysis showed that the blank group, model group and GHC group were obviously separated, and the samples in the group were aggregated, indicating that the determined metabolites changed significantly among the three groups. P-test analysis showed that the separation models between different groups were not over-fitted (Figure 11).

**FIGURE 12:**
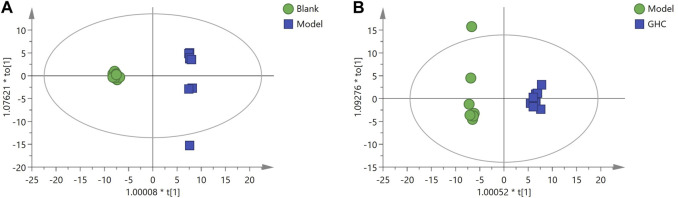
OPLS-DA analysis of serum metabolites in the treatment of acute alcoholic liver injury with *Penthorum chinense* Pursh. **(A)** Blank group vs. Model group; **(B)** Model group vs. GHC group.

OPLS-DA analysis was performed to screen differential metabolites (Figure 12). The results showed that the blank group, model group and GHC group could be separated obviously. The greater the VIP value, the greater the contribution of the difference between groups (Supplementary Tables S2, S3).

According to the variable importance in projection (VIP) of the constructed OPLS-DA model, potential biomarkers were screened under the condition of VIP > 1 and *p* < 0.05, and 50 potential biomarkers in serum were screened out (Table 1).

**TABLE 1:** Trends of potential biomarkers in serum.

ID	Model vs. blank	Model vs. GHC
SM C16:1	↑↑	↓↓
Uridine	↑↑	↓↓
Erythrose4-phosphate	↑↑	↓↓
And	↑↑	↓
GDCA	↓↓	↑↑
lysoPC a C24:0	↑↑	↓↓
lysoPC a C18:1	↑↑	↓↓
8-iso-PGF	↑↑	↓↓
Aminohippuric acid	↑↑	↓↓
Cortisol	↑↑	↓↓
TCA	↑↑	↓↓
FA (18:2)	↑↑	↓↓
ACO	↑↑	↓
TLCA	↓↓	↑↑
GCDCA	↓↓	↑↑
C18:0LPC	↑↑	↓
ALL	↑↑	↓↓
EPA [FA (20:5ω3)]	↑↑	↓↓
Met	↑↑	↓↓
C8	↓↓	↑
FA (20:2)	↑↑	↓↓
DHA [FA (22:6ω3)]	↑↑	↓↓
HArg	↓↓	↑
C18	↑↑	↓↓
Carnosine	↑↑	↓
AA [FA (20:4ω6)]	↑↑	↓↓
C18:1LPC	↑↑	↓↓
C16:0LPC	↑↑	↓
FA (16:0)	↑↑	↓↓
C14:2	↑↑	↓
GUDCA	↓↓	↑
Xanthine	↑↑	↓
Opht A	↑↑	↓↓
Palmitoyl-L-carnitine	↑↑	↓↓
FA (20:3)	↑↑	↓↓
I-Tyr	↑↑	↓↓
Choline	↑↑	↓↓
2-DG	↑↑	↓↓
FA (18:1)	↑	↓
lysoPC a C18:2	↑↑	↓↓
17-HYD	↑↑	↓↓
3-IAA	↑	↓↓
C18:1	↑↑	↓
GABA	↑↑	↓↓
4,6-dihydroxyquinoline	↑	↓
Carnitine	↑	↓
AbsAcid	↓↓	↑↑
lysoPC a C16:0	↑↑	↓↓
lysoPC a C18:0	↑	↓
Spermidine	↑↑	↓↓

Note: ↑ means that the average content of metabolites increased *p* < 0.05, ↑↑ means that the average content of metabolites increased *p* < 0.01, ↓ means that the average content of metabolites decreased *p* < 0.05, ↓↓ means that the average content of metabolites decreased *p* < 0.01.

Note: ↑ means that the average content of metabolites increased *p* < 0.05, ↑↑ means that the average content of metabolites increased *p* < 0.01, ↓ means that the average content of metabolites decreased *p* < 0.05, ↓↓ means that the average content of metabolites decreased *p* < 0.01.

**FIGURE 13:**
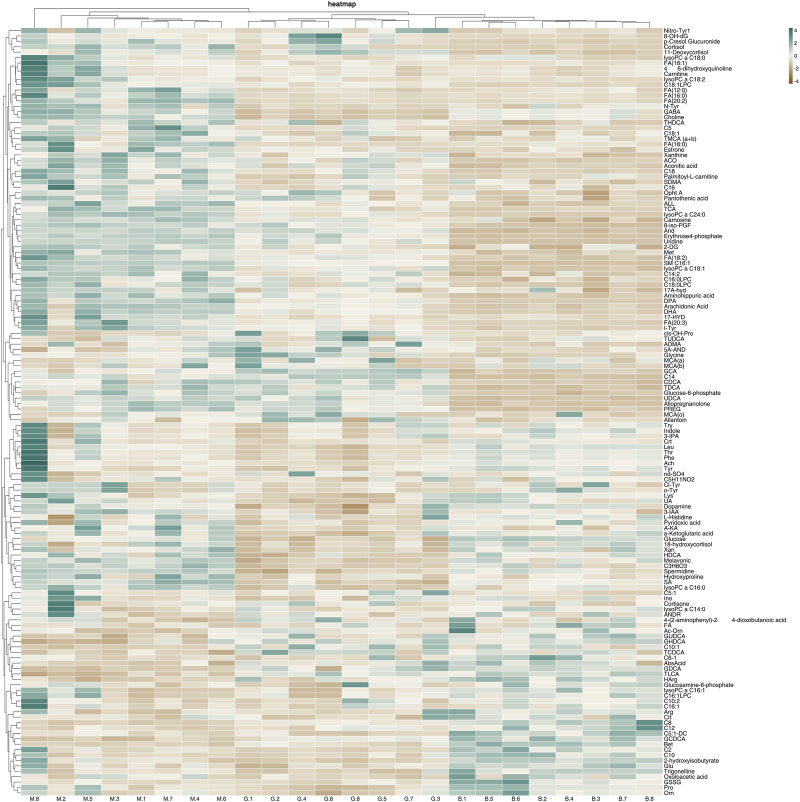
Heat map of metabolites in serum of mice with acute alcoholic liver injury.

In order to further understand the metabolic differences between different groups, 142 metabolites in serum were blankized by Metaboanalyst website to generate a cluster heat map (Figure 13).

**FIGURE 14:**
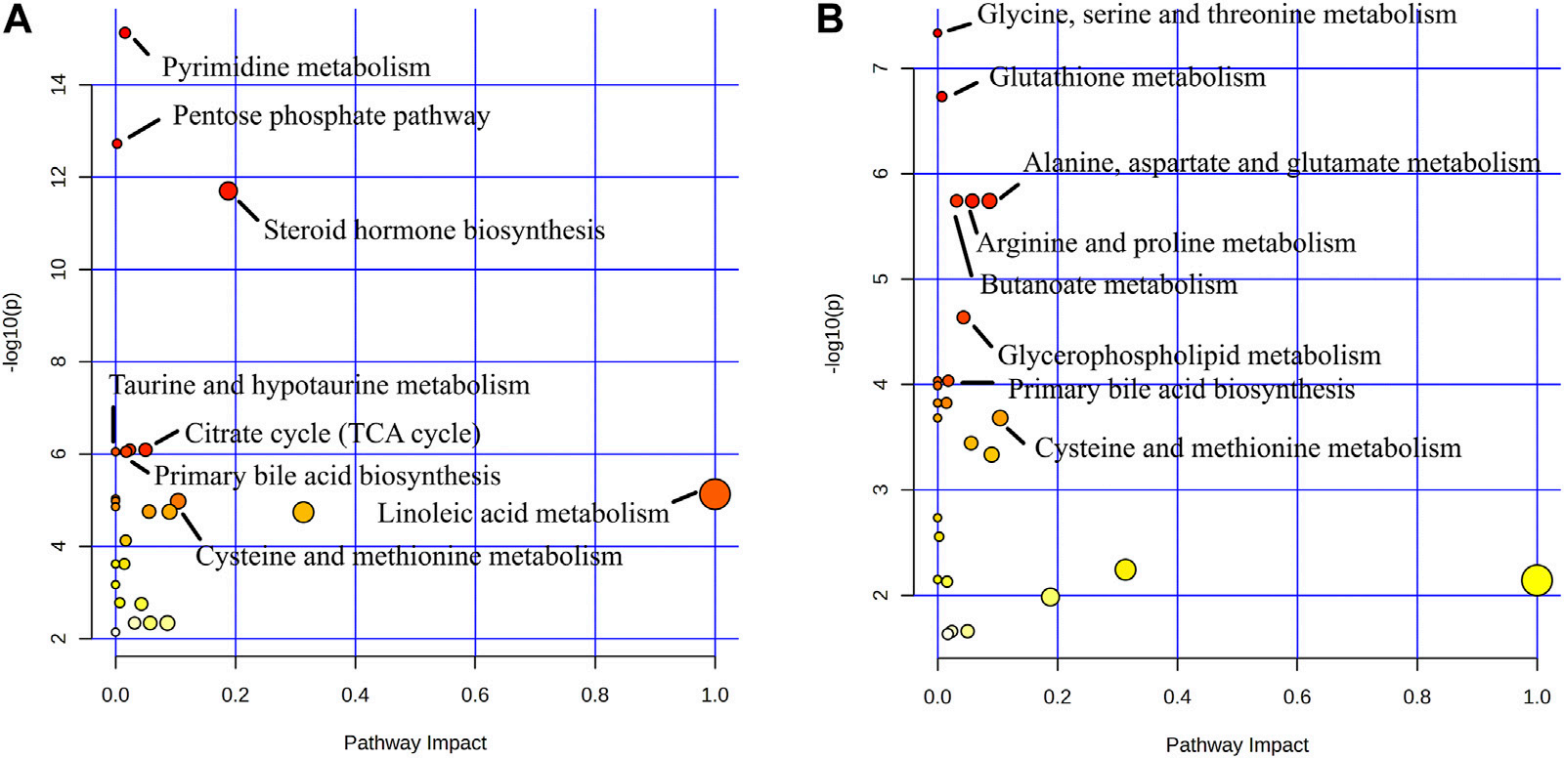
Metabolite pathway analysis. **(A)** Blank group vs. Model group; **(B)** Model group vs. GHC group.

In order to explore the potential metablic pathways of GHC on the treatment of ALD in mice, all differential metabolites were imported into the Metaboanalyst 5.0 online analysis platform to find their correlation, and the metabolic pathways most related to the treatment of ALD by GHC were screened for metabolic pathway analysis (Figure 14).

Metabolic pathway analysis revealed important metabolic pathways related to ALD including Pyrimidine metabolism, Pentose phosphate pathway, Steroid hormone biosynthesis, Citrate cycle (TCA cycle), Glyoxylate and dicarboxylate metabolism, Primary bile acid biosynthesis and Taurine and hypotaurine metabolism. After administration, it mainly affected Glycine, serine and threonine metabolism, Glutathione metabolism, Arginine and proline metabolism, Alanine. Metabolic pathways such as aspartate and glutamate metabolism, butanoate metabolism and primary bile acid biosynthesis.

## 4 Discussion

ALD is a common liver disease caused by long-term drinking. At present, the main intervention methods are alcohol withdrawal and drug combination therapy, but there is still a lack of effective drugs. In this study, ALT, AST, TC and TG activities in serum of mice were increased after alcohol-induced ALD model in mice, and the liver index of mice in the model group was significantly increased, indicating that liver tissue suffered damage. After treatment with *Penthorum chinense* Pursh, content of ALT, AST, TC and TG in serum and liver index decreased. It shows that *Penthorum chinense* Pursh has a good therapeutic effect on ALD in mice.

The gut-liver axis plays an important role in the development of ALD. Changes in the structure of intestinal flora and the entry of bacterial products into the blood circulation play an important role in ALD (Vieira-Silva et al., 2020; Yang et al., 2020). Intestinal microecological imbalance can be manifested as quantitative changes in intestinal microflora and qualitative changes (Chen et al., 2011; Leclercq et al., 2014). The relative abundance of Firmicutes in the intestine of patients with cirrhosis caused by ALD increased significantly, and the relative abundance of Bacteroidetes decreased significantly (Adawi et al., 2001). In this study, compared with the blank group, the relative abundance of Firmicutes in the model group increased, but the relative abundance of Bacteroidetes decreased. GHC could significantly reversed the changes of Firmicutes and Bacteroidetes. The increased abundance of *Deferribacteres* in the gut could enhance the regulate ability of the immune system (Ma et al., 2019). Drinking could led to a significant decrease in the relative abundance of *Deferribacteres* in the intestinal flora, while the GHC could increased the relative abundance of *Deferribacteres* in the intestine. *Odoribacter* could produce isoallocholic acid (isoalloLCA, a new type of bile acid). IsoalloLCA can enhance the differentiation of T cells by promoting the formation of the allowed chromatin structure in the Foxp3 promoter region. In this study, the abundance of *Odoribacter* in the intestine of the model group was reduced. After treatment with GHC, the abundance of *Odoribacter* increased significantly.

Metabolites produced by symbiotic bacteria may also be involved in the pathogenesis of ALD. Under stimulation, the human intestinal flora can produce bioactive compounds such as bile acids, short-chain fatty acids, ammonia, phenols, and endotoxin to reduce or aggravate liver steatosis and inflammation (Lee and Jayaraman, 2019). Therefore, targeted metabolomics were used to analyszed the mechanism of therapeutic effect on ALD. In this study, metabolic pathways such as Pyrimidine metabolism, Pentose phosphate pathway, Steroid hormone biosynthesis, Citrate cycle (TCA cycle), Glyoxylate and dicarboxylate metabolism, Primary bile acid biosynthesis and Taurine and hypotaurine metabolism were closely related to the pathogenesis of ALD. GHC play a therapeutic role by regulating the pathway of Glycine, serine and threonine metabolism, Glutathione metabolism, Arginine and proline metabolism, Alanine, aspartate and glutamate metabolism, Butanoate metabolism and primary bile acid biosynthesis.

Cholestasis is one of the key pathogenic factors of ALD. Long-term alcohol consumption can lead to abnormal changes in the size and composition of bile acid pools, which in turn may affect ALD (Liu et al., 2022b). Therefore, regulating bile acid metabolism was considered as a potential therapeutic strategy for ALD. Intestinal flora plays an important role in bile acid metabolism by participating in deadhesion, oxidation, differential isomerization, 7α-dehydrogenation, esterification and desulfurization (Gnewuch et al., 2009). ALD leads to intestinal flora imbalance, bile acid metabolism disorder. After treatment with GHC, bile acid metabolism returned to normal, indicating that GHC can treat ALD by regulating the metabolic pathway of primary bile acid biosynthesis.

When the liver is damaged, amino acid metabolism *in vivo* could be disordered which will affect the physiological function of the liver (Zhang and Zhang, 2016). In this study, the results showed that the amino acid metabolism in the serum of ALD mice was disordered. After treatment with GHC, most amino acids recalled, compared with that in ALD mice.

Glutathione is a tripeptide composed of glutamic acid, cysteine and glycine. Glutathione, especially glutathione in liver cells, can participate in biotransformation, converting harmful materials substances including alcohol into harmless substances and excreting them out of the body (Zira et al., 2013). Glutathione decreased significantly in liver disease such as alcoholic liver disease, hepatic veno-occlusive disease, chronic hepatitis C and Wilson’s disease.

## 5 Conclusion

GHC could improve the ALD mice by regulating the imbalance of intestinal flora as well as regulating the metabolic pathways such as Glycine, serine and threonine metabolism, Glutathione metabolism, Arginine and proline metabolism, Alanine, aspartate and glutamate metabolism, Butanoate metabolism and Primary bile acid biosynthesis to treat ALD.

## Data Availability

The datasets presented in this study can be found in online repositories. The names of the repository/repositories and accession number(s) can be found below: https://doi.org/10.5281/zenodo.10260384.
