# Supplementary material for: Regulation of intestinal microflora and metabolites of Penthorum chinense Pursh on alcoholic liver disease
Source: Front Pharmacol. 2024 Jan 24;14:1331956. doi: 10.3389/fphar.2023.1331956 (PMC10847573; doi:10.3389/fphar.2023.1331956)
Supplement: Supplementary file 1 [file DataSheet1.docx]

Supplementary Material

Regulation of intestinal microecology and metabolic mechanism of *Penthorum chinense* Pursh in the treatment of alcoholic liver disease

Hui Zhang^1^, Xiao Cui^1^, Wei Liu^2^, Zheng Xiang^2*^ and Ji-Feng Ye^1*^,

^1^Department of Pharmacy, the Second Affiliated Hospital and Yuying Children's Hospital of Wenzhou Medical University, Wenzhou, China

^2^School of Pharmaceutical Science, Liaoning University, Shenyang, China

*** Correspondence:**Zheng Xiang,
rainbowaftersnow@hotmail.com

Ji-Feng Ye,
[yjfWZM@163.com](mailto:yjfWZM@163.com)

# Supplementary Figures and Tables

## Supplementary Figures


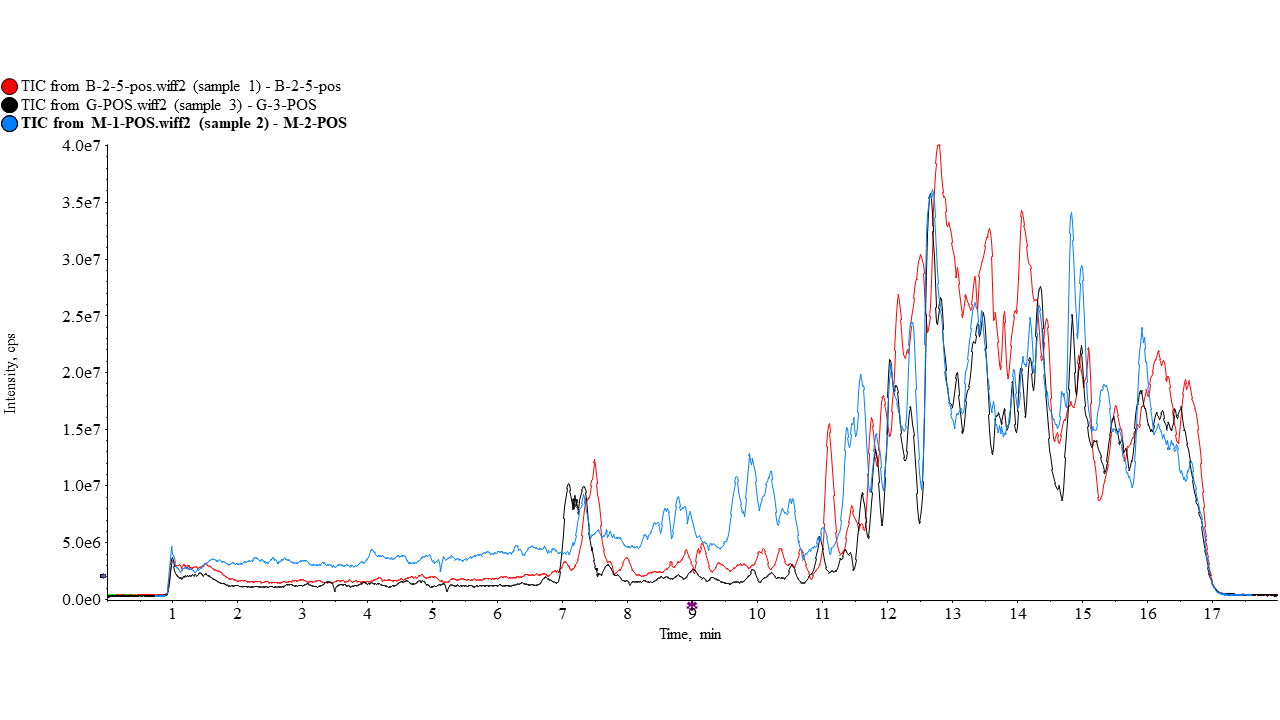


A. Total ion chromatogram of the metabolites in the serum in the positive ion mode


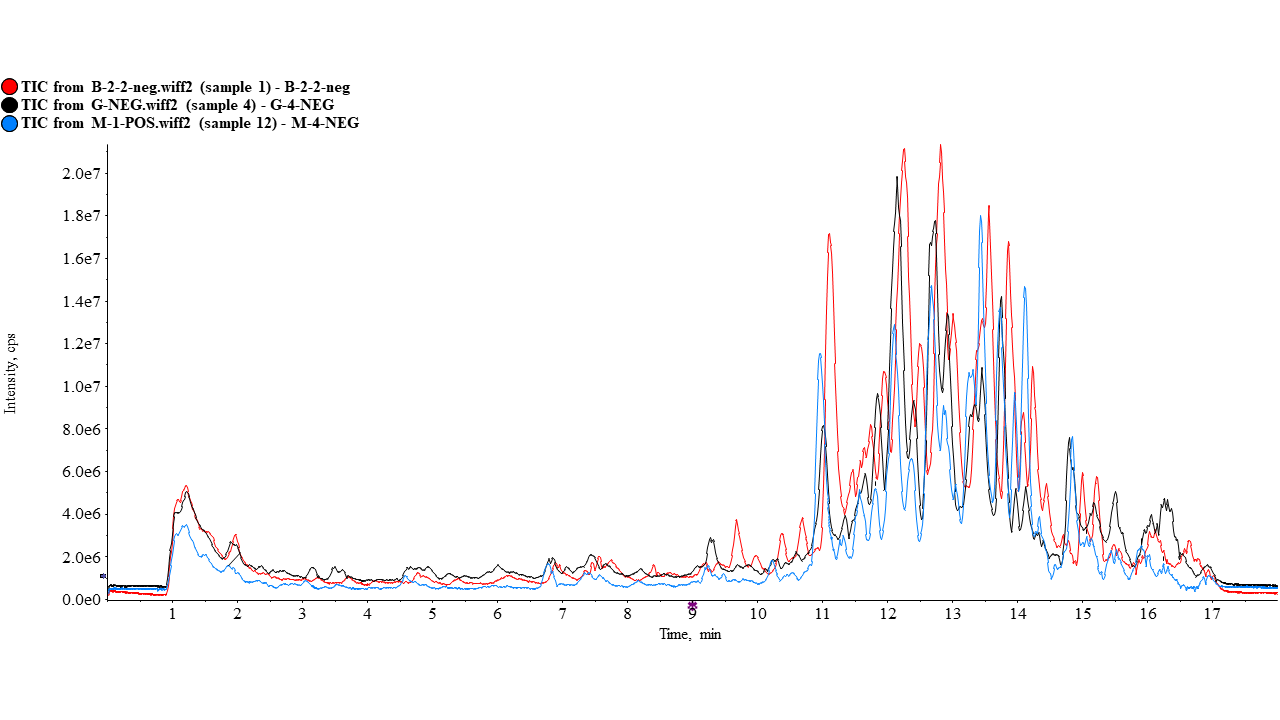


B. Total ion chromatogram of the metabolites in the serum in the negative ion mode

**Supplementary Figure 1.** Total ion chromatogram of each biomarker in serum

## Supplementary Tables

**Supplementary Table 1.** Compound excimer ion and fragment information.

| **Peak** | **Analyte** | ***R_t_***  **(min)** | **[M-H]-/ [M+HCOO]‒**  **(m/z)** | **Fragmentary ions** | **[M+H]+/ [M+Na]+/ [M+K]+**  **(m/z)** | **Fragmentary ions** | **Formula** |
| --- | --- | --- | --- | --- | --- | --- | --- |
| **1** | Chebulic acid | 2.690 | 355.0311 | 337.0207, 293.0487 | - | - | C_14_H_12_O_11_ |
| **2** | Gallic acid | 4.697 | 169.0137 | 151.0085 | 171.0284 | 153.0180, 109.0225 | C_7_H_6_O_5_ |
| **3** | Protocatechuic acid | 10.077 | 153.0187 | 109.0035, 135.0061 | - | - | C_7_H_6_O_4_ |
| **4** | Methyl gallate | 18.174 | 183.0294 | 165.0161, 139.0390 | - | - | C_8_H_8_O_5_ |
| **5** | Catechin | 20.235 | 289.0719 | 245.0629, 178.9781 | 291.0862 | 273.0763 | C_15_H_14_O_6_ |
| **6** | Rutin | 26.967 | 609.1456 | 300.8537, 463.0840 | - | - | C_27_H_30_O_16_ |
| **7** | Vanillic acid | 27.368 | 167.0344 | 152.0473, 139.0366 | 169.0492 | 141.1132 | C_8_H_8_O_4_ |
| **8** | 2-(Beta-D-Glucopyranosyloxy)Benzaldehyde | 27.466 | 329.0875 | 121.0686 | - | - | C_13_H_16_O_7_ |
| **9** | 2,6-Dihydroxyacetophenone-4-O-β-D-glucopyranoside | 27.326 | 329.0875 | 166.9269, 123.9022 | 331.1025 | 169.0495 | C_14_H_18_O_9_ |
| **10** | Ethyl gallate | 30.518 | 197.0451 | 168.0371, 178.9784 | - | - | C_9_H_10_O_5_ |
| **11** | Ellagic acid | 38.671 | 300.9985 | 265.0085 | - | - | C_14_H_6_O_8_ |
| **12** | Isoquercitrin | 41.244 | 463.0885 | 178.9758, 301.0694 | 465.1033 | 303.0501, 182.9812 | C_21_H_20_O_12_ |
| **13** | Quercetin-3-O-xyloside | 50.260 | 433.0778 | 283.0249, 301.0360 | 435.0925 | 303.0502 | [C_20_H_18_O_11_](https://pubchem.ncbi.nlm.nih.gov/#query=C20H18O11) |
| **14** | Cynaroside | 52.614 | 447.0924 | 285.0790 | 449.1084 | 287.0555, 193.0882 | C_21_H_20_O_11_ |
| **15** | Zingiberin-7-O-glucoside | 57.050 | 477.1392 | 315.0259 | 433.1493 | 271.0954 | C_22_H_24_O_9_ |
| **16** | Kaempferol-3-O-arabinoside | 58.110 | 417.0823 | 285.0361, 257.0425 | 419.0972 | 287.0545, 167.0129 | C_20_H_18_O_10_ |
| **17** | Apigenin-7-O-β-D-glucoside | 59.471 | 431.0984 | 268.9979, 250.9875 | 433.1132 | 271.1925 | C_21_H_20_O_10_ |
| **18** | Pinocembrin-7-O-β-D-glucoside | 68.719 | 417.1194 | 255.0021, 236.9902 | 419.1339 | 257.0775 | C_21_H_22_O_9_ |
| **19** | 2',4',6'-trihydroxydihydrochalcone-4'-β-D-glucoside | 69.779 | 419.1344 | 256.9947, 136.9220 | - | - | C_21_H_24_O_9_ |
| **20** | Pinocembrin-7-O- (4',6'-hexahydroxydibenzoyl) -β-glucoside | 78.528 | 719.1243 | 254.9895 | 721.1407 | 257.0810, 163.0360 | C_35_H_28_O_17_ |
| **21** | 2',6'-Dihydroxydihydrochalcone-4'-O- (3','-O-galloyl) -β-D-glucopyranoside | 82.541 | 571.1462 | 419.0030, 257.0091 | - | - | C_28_H_28_O_13_ |
| **22** | Quercetin-3-O-β-D-xylopyranosyl- (1→2) -β-D-galactopyranoside | 82.792 | 595.1311 | 301.1225, 433.0022 | - | - | [C_26_H_28_O_16_](https://pubchem.ncbi.nlm.nih.gov/#query=C26H28O16) |
| **23** | Thonningianin B | 83.184 | 721.1408 | 419.1308 | 723.1564 | 421.1463 | C_35_H_30_O_17_ |
| **24** | Pinocembrin-7-O- (3'-O-galloyl-4',6'-hexahydroxydibenzoyl) -β-glucoside | 83.868 | 871.1359 | 417.0178 | 873.1517 | 257.0809 | C_42_H_32_O_21_ |
| **25** | Penthorumine C | 84.937 | 341.1024 | 313.0996, 268.9945 | - | - | C_19_H_18_O_6_ |
| **26** | ThonningianinA | 84.912 | 873.1502 | 571.1395 | 897.1477 | 595.3300 | C_42_H_34_O_21_ |
| **27** | Penthorumin D | 88.501 | 311.0917 | 293.0915, 265.0839 | 313.1066 | 285.1774 | C_18_H_16_O_5_ |
| **28** | Penthionone C | 89.675 | 325.1072 | 307.1111, 133.2510 | - | - | C_19_H_18_O_5_ |
| **29** | Quercetin-3-O-acacetin-7-O-glucoside | 27.596 | 771.1987 | 609.1443, 463.0625 | 773.2136 | 611.1587 | C_33_H_40_O_21_ |
| **30** | Quercetin-3-O-robinobioside | 36.1 | - | - | 611.1611 | 303.0530, 209.0830 | C_27_H_30_O_16_ |
| **31** | Kaempferol-3-O-rutinoside | 31.461 | 593.1516 | 447.1286, 285.0442 | 595.1666 | 287.1485 | C_27_H_30_O_15_ |
| **32** | Quercetin-5,7-O-diglucoside | 32.220 | 625.1408 | 463.0878, 301.0005 | 627.1560 | 465.1039, 303.0325 | C_27_H_30_O_17_ |
| **33** | Scopolamine | 37.470 | - | - | 193.0497 | 162.0565 | C_10_H_8_O_4_ |
| **34** | Quercetin-3-O-arabinoside | 44.757 | 433.0770 | 300.9989, 180.9862 | 473.0478 | 340.9945, 246.9778 | C_20_H_18_O_11_ |
| **35** | Quercetin | 52.586 | 447.0926 | 300.9842 | 449.1084 | 303.0505 | C_21_H_20_O_11_ |
| **36** | Luteolin | 58.195 | - | - | 287.0550 | 279.1008, 167.0140 | C_15_H_10_O_6_ |
| **37** | Baicalein-7-O-rhamnoside | 59.622 | - | - | 433.1134 | 287.0551, 182.1422 | C_21_H_20_O_10_ |
| **38** | Pinocembranone chalcone | 68.637 | 255.0666 | 227.9326, 161.0262 | 257.0808 | 213.0722 | [C_15_H_12_O_4_](https://pubchem.ncbi.nlm.nih.gov/#query=C15H12O4) |

*R_t_*, retention times. [M-H]-/ [M+HCOO]^‒^, pseudomolecular ions in MS spectra recorded in a negative mode. [M+H]+/ [M+Na]+/ [M+K]^+^, pseudomolecular ions in MS spectra recorded in a positive mode.

**Supplementary Table 2.** The VIP values of serum metabolites in normal group and model group were compared.

| Var ID (Primary) | VIP |
| --- | --- |
| SM C16:1 | 1.39272 |
| Uridine | 1.39079 |
| Erythrose4-phosphate | 1.38846 |
| And | 1.38152 |
| GDCA | 1.37136 |
| lysoPC a C24:0 | 1.36843 |
| UDCA | 1.36788 |
| lysoPC a C18:1 | 1.36046 |
| 8-iso-PGF | 1.35011 |
| Allopregnanolone | 1.33635 |
| Aminohippuric acid | 1.31305 |
| C14 | 1.31053 |
| Bet | 1.30946 |
| TDCA | 1.30645 |
| Cortisol | 1.30271 |
| TCA | 1.29339 |
| FA(18:2) | 1.28822 |
| PREG | 1.28429 |
| ACO | 1.28118 |
| TLCA | 1.27714 |
| GCDCA | 1.27005 |
| C18:0LPC | 1.26558 |
| ALL | 1.26012 |
| EPA（FA（20：5ω3）) | 1.25153 |
| Met | 1.23317 |
| C8 | 1.2292 |
| FA(20:2) | 1.21895 |
| DHA（FA（22：6ω3）） | 1.21645 |
| HArg | 1.21645 |
| C18 | 1.20811 |
| 8-OH-dG | 1.20255 |
| Aconitic acid | 1.20188 |
| Carnosine | 1.20154 |
| C5:1-DC | 1.19467 |
| AA（FA（20:4ω6）) | 1.19009 |
| p-Cresol Glucuronide | 1.19006 |
| C18:1LPC | 1.18924 |
| C16:0LPC | 1.18417 |
| FA(16:0) | 1.18129 |
| C14:2 | 1.18014 |
| GUDCA | 1.17255 |
| 11-Deoxycortisol | 1.17081 |
| Xanthine | 1.16486 |
| Glucose-6-phosphate | 1.15313 |
| Opht A | 1.15038 |
| C5 | 1.14314 |
| THDCA | 1.14087 |
| Palmitoyl-L-carnitine | 1.13947 |
| FA(20:3) | 1.12597 |
| I-Tyr | 1.12225 |
| Pantothenic acid | 1.11915 |
| SA | 1.11841 |
| Choline | 1.1103 |
| CDCA | 1.10957 |
| 2-DG | 1.1088 |
| C10 | 1.10666 |
| Glucose | 1.09748 |
| FA(18:1) | 1.0898 |
| GCA | 1.08925 |
| lysoPC a C18:2 | 1.08007 |
| 17-HYD | 1.06931 |
| 2-hydroxyisobutyrate | 1.06888 |
| 3-IAA | 1.06168 |
| C18:1 | 1.06082 |
| GABA | 1.05881 |
| C12 | 1.0359 |
| FA(18:0) | 1.03476 |
| 4,6-dihydroxyquinoline | 1.0331 |
| Carnitine | 1.033 |
| Glycine | 1.03071 |
| AbsAcid | 1.02218 |
| lysoPC a C16:0 | 1.01785 |
| lysoPC a C18:0 | 1.01216 |
| Spermidine | 1.00259 |
| Trigonelline | 0.996017 |
| GSSG | 0.994677 |
| GHDCA | 0.988059 |
| Estrone | 0.986323 |
| C6-1 | 0.975222 |
| FA(12:0) | 0.970498 |
| SDMA | 0.965423 |
| TCDCA | 0.964888 |
| Glu | 0.949515 |
| TMCA (a+b) | 0.945204 |
| C10:1 | 0.938746 |
| 17A-hyd | 0.930402 |
| N-Tyr | 0.929357 |
| C3H6O3 | 0.910842 |
| A-KA | 0.907431 |
| Hydroxyproline | 0.904145 |
| C2 | 0.893968 |
| lysoPC a C16:1 | 0.883227 |
| C10:2 | 0.872131 |
| Nitro-Tyr1 | 0.87019 |
| Arg | 0.868933 |
| Phe | 0.863692 |
| Tyr | 0.861119 |
| Thr | 0.853537 |
| C16 | 0.844623 |
| Ac-Orn | 0.836101 |
| Pro | 0.823957 |
| Melavonic | 0.812924 |
| Ach | 0.812066 |
| FA | 0.807598 |
| C16:1LPC | 0.802176 |
| Crt | 0.799942 |
| C16:1 | 0.781694 |
| Lys | 0.781576 |
| Leu | 0.780955 |
| ADMA | 0.741264 |
| Indole | 0.706203 |
| Glucosamine-6-phosphate | 0.681849 |
| 5A-AND | 0.676342 |
| Cit | 0.675005 |
| C5:1 | 0.673625 |
| Orn | 0.672452 |
| 3-IPA | 0.670483 |
| Try | 0.661749 |
| 4-(2-aminophenyl)-2,4-dioxobutanoic acid | 0.58465 |
| Oxoloacetic acid | 0.571042 |
| nd-SO4 | 0.567023 |
| Dopamine | 0.562364 |
| cis-OH-Pro | 0.552783 |
| Allantoin | 0.524434 |
| C5H11NO2 | 0.515531 |
| Cortisone | 0.499081 |
| TUDCA | 0.47918 |
| a-Ketoglutaric acid | 0.406947 |
| ANDR | 0.404206 |
| Ino | 0.402106 |
| MCA(b) | 0.392723 |
| HDCA | 0.380196 |
| MCA(a) | 0.373792 |
| UA | 0.368955 |
| MCA(o) | 0.342983 |
| lysoPC a C14:0 | 0.335258 |
| 18-hydroxycortisol | 0.33269 |
| L-Histidine | 0.329559 |
| Pyridoxic acid | 0.288456 |
| Cl-Tyr | 0.286746 |
| o-Tyr | 0.257673 |
| Xan | 0.116732 |

**Supplementary Table 3.** The VIP values of serum metabolites in normal group and model group were compared.

| Var ID (Primary) | VIP |
| --- | --- |
| SM C16:1 | 1.54939 |
| Choline | 1.50981 |
| C3H6O3 | 1.49718 |
| C8 | 1.489 |
| GABA | 1.47441 |
| Melavonic | 1.47355 |
| Spermidine | 1.45623 |
| lysoPC a C24:0 | 1.44764 |
| SA | 1.42664 |
| FA(20:2) | 1.39865 |
| TLCA | 1.37129 |
| FA(16:0) | 1.36704 |
| lysoPC a C16:0 | 1.36585 |
| TCA | 1.35656 |
| GCA | 1.34711 |
| Glucose | 1.3292 |
| GCDCA | 1.32485 |
| N-Tyr | 1.31922 |
| Carnosine | 1.28629 |
| Met | 1.28177 |
| 2-DG | 1.26292 |
| FA(18:0) | 1.25052 |
| ALL | 1.24607 |
| lysoPC a C18:1 | 1.2448 |
| Aminohippuric acid | 1.23779 |
| 17-HYD | 1.22573 |
| C10:1 | 1.22351 |
| FA(12:0) | 1.2127 |
| TCDCA | 1.21264 |
| AbsAcid | 1.20423 |
| HDCA | 1.196 |
| FA(18:2) | 1.18771 |
| Xan | 1.18554 |
| Erythrose4-phosphate | 1.18059 |
| DHA（FA（22：6ω3）） | 1.17936 |
| GDCA | 1.17143 |
| Leu | 1.17129 |
| EPA（FA（20：5ω3）) | 1.17124 |
| C18:1LPC | 1.16527 |
| lysoPC a C18:0 | 1.16511 |
| Palmitoyl-L-carnitine | 1.16413 |
| FA(18:1) | 1.16206 |
| Thr | 1.15481 |
| C5 | 1.14704 |
| SDMA | 1.1463 |
| Phe | 1.14626 |
| 2-hydroxyisobutyrate | 1.14044 |
| 17A-hyd | 1.13197 |
| 4,6-dihydroxyquinoline | 1.1216 |
| Carnitine | 1.11977 |
| lysoPC a C18:2 | 1.10436 |
| Hydroxyproline | 1.0915 |
| Crt | 1.08315 |
| A-KA | 1.08209 |
| FA(20:3) | 1.07935 |
| C2 | 1.07472 |
| Uridine | 1.064 |
| HArg | 1.04564 |
| AA（FA（20:4ω6）) | 1.04339 |
| C18 | 1.04242 |
| GHDCA | 1.03944 |
| Cortisol | 1.0374 |
| Pro | 1.03649 |
| C16:0LPC | 1.03397 |
| Bet | 1.02604 |
| C14:2 | 1.02469 |
| Lys | 1.01757 |
| Ach | 1.01751 |
| 8-iso-PGF | 1.01456 |
| I-Tyr | 1.01062 |
| Estrone | 1.00516 |
| 3-IAA | 1.00116 |
| GUDCA | 0.99828 |
| Allopregnanolone | 0.997558 |
| Tyr | 0.989799 |
| a-Ketoglutaric acid | 0.985464 |
| UA | 0.973561 |
| And | 0.953268 |
| Pyridoxic acid | 0.949197 |
| 5A-AND | 0.946778 |
| Dopamine | 0.944033 |
| C16:1 | 0.943629 |
| Ac-Orn | 0.936269 |
| C18:0LPC | 0.936048 |
| C18:1 | 0.934464 |
| PREG | 0.931531 |
| C14 | 0.913931 |
| Orn | 0.911213 |
| Glu | 0.899438 |
| Indole | 0.892694 |
| Oxoloacetic acid | 0.881664 |
| ACO | 0.881237 |
| 18-hydroxycortisol | 0.870577 |
| 3-IPA | 0.868643 |
| C16:1LPC | 0.866171 |
| Xanthine | 0.864024 |
| lysoPC a C16:1 | 0.856427 |
| Try | 0.85516 |
| C10 | 0.849397 |
| Trigonelline | 0.847347 |
| TMCA (a+b) | 0.845891 |
| C16 | 0.837036 |
| cis-OH-Pro | 0.823372 |
| o-Tyr | 0.805324 |
| Cl-Tyr | 0.790873 |
| C10:2 | 0.766607 |
| C12 | 0.764094 |
| Allantoin | 0.745802 |
| Glycine | 0.690991 |
| THDCA | 0.690209 |
| C5H11NO2 | 0.685198 |
| 4-(2-aminophenyl)-2,4-dioxobutanoic acid | 0.672005 |
| Aconitic acid | 0.648946 |
| Nitro-Tyr1 | 0.641894 |
| Ino | 0.6367 |
| Arg | 0.621235 |
| nd-SO4 | 0.603781 |
| MCA(a) | 0.586111 |
| MCA(o) | 0.585349 |
| C5:1-DC | 0.578013 |
| Opht A | 0.562886 |
| GSSG | 0.538417 |
| Glucose-6-phosphate | 0.523418 |
| Pantothenic acid | 0.502794 |
| TUDCA | 0.486355 |
| ADMA | 0.440187 |
| TDCA | 0.401247 |
| MCA(b) | 0.397974 |
| CDCA | 0.363344 |
| FA | 0.362587 |
| 8-OH-dG | 0.329566 |
| UDCA | 0.30998 |
| C6-1 | 0.308025 |
| ANDR | 0.268374 |
| lysoPC a C14:0 | 0.213803 |
| L-Histidine | 0.208753 |
| Glucosamine-6-phosphate | 0.197702 |
| p-Cresol Glucuronide | 0.192661 |
| C5:1 | 0.182803 |
| 11-Deoxycortisol | 0.160702 |
| Cit | 0.0811475 |
| Cortisone | 0.0482672 |
